# Association of High-Intensity Binge Drinking With Lipid and Liver Function Enzyme Levels

**DOI:** 10.1001/jamanetworkopen.2019.5844

**Published:** 2019-06-14

**Authors:** Daniel B. Rosoff, Katrin Charlet, Jeesun Jung, Jisoo Lee, Christine Muench, Audrey Luo, Martha Longley, Kelsey L. Mauro, Falk W. Lohoff

**Affiliations:** 1 Section on Clinical Genomics and Experimental Therapeutics, National Institute on Alcohol Abuse and Alcoholism, National Institutes of Health, Bethesda, Maryland; 2 Department of Psychiatry and Psychotherapy, Charite–Universitaetsmedizin Berlin, Berlin, Germany

## Abstract

**Importance:**

The prevalence of high-intensity binge drinking (HIBD), defined as consuming 2 or more times the binge threshold defined by the National Institute on Alcohol Abuse and Alcoholism (NIAAA), is rapidly increasing in the United States. While the relationship between alcohol consumption and lipid and liver function enzyme (LFT) biomarkers has been previously examined, the associations of HIBD with those biomarkers remain unknown.

**Objective:**

To examine associations of HIBD with lipid and LFT levels in a cross-sectional sample enriched with participants who engage in HIBD.

**Design, Setting, and Participants:**

Cross-sectional study using data from the NIAAA clinical sample collected from March 3, 2005, to August 21, 2017, with participants recruited for either the NIAAA screening protocols or inpatient alcohol treatment program. For this study, participants were stratified by self-reported alcohol consumption into 4 sex-specific binge levels: nonbinge and 1, 2, and 3 or more times the binge threshold (levels I, II, and III). Multivariable analyses examined the odds of clinically high levels of lipids and LFTs across binge levels. Analyses were performed from December 3, 2018, to January 30, 2019.

**Main Outcomes and Measures:**

Serum levels of high-density lipoprotein cholesterol (HDL-C), low-density lipoprotein cholesterol, total cholesterol, triglycerides, alanine aminotransferase, aspartate aminotransferase, and γ-glutamyltransferase.

**Results:**

A total of 2065 participants underwent protocol screening; 1519 with data available on alcohol consumption, body mass index, lipid levels, and LFT levels were included in the final analyses. Mean (SD) age was 39.7 (12.1) years; mean (SD) body mass index was 26.6 (5.1); 978 (64.4%) were male; 718 (47.3%) were white; and 578 (31.1%) consumed alcohol at the nonbinge level, 321 (21.2%) at level I, 239 (15.7%) at level II, and 318 (25.1%) at level III. High-intensity binge drinking was associated with 2- to 8-fold increased odds for clinically high levels of HDL-C, total cholesterol, triglycerides, and all LFTs (eg, for HDL-C: level III odds ratio [OR], 8.65; 95% CI, 4.75-15.77 and for γ-glutamyltransferase: level III OR, 8.21; 95% CI, 5.90-11.43). Increased HIBD frequency (days consuming at levels II and III) was associated with increased odds for clinically high levels of HDL-C, total cholesterol, and all LFTs (per unit increase in days consuming at the respective binge level) (eg, for HDL-C: level II OR, 1.025; 95% CI, 1.014-1.036 and level III OR, 1.033; 95% CI, 1.019-1.047 and for γ-glutamyltransferase: level II OR, 1.028; 95% CI, 1.019-1.037 and level III OR, 1.033; 95% CI, 1.019-1.047).

**Conclusions and Relevance:**

High-impact binge drinking was significantly associated with increased odds for clinically high levels of lipids and LFTs. Given that HIBD is increasingly common among US adults, targeted interventions aimed at reducing HIBD may have important health benefits.

## Key Points

**Question:**

What changes in circulating lipid and liver function enzyme levels are associated with high-intensity binge drinking?

**Findings:**

In this cross-sectional study of 1519 participants, high-intensity binge drinking was associated with increased cholesterol, triglyceride, and liver function enzyme levels.

**Meaning:**

Lipid and liver function enzyme levels demonstrate dose-dependent increases with high-intensity binge drinking, indicating potential adverse health outcomes may be associated with such drinking behavior.

## Teaser

This cohort study examines the associations of high-intensity binge drinking with changes in levels of serum lipids and liver function enzymes.

## Introduction

The percentage of US adults reporting binge drinking, defined by the National Institute on Alcohol Abuse and Alcoholism (NIAAA) as 4 or more drinks for women and 5 or more drinks for men in a given day,^1^ has escalated in the last 2 decades, with recent studies^2,3^ finding that approximately 32 million adults engage in high-intensity binge drinking (HIBD), defined as consuming 2 or more times the NIAAA binge drinking cutoff.^4^ This trend suggests that negative health outcomes associated with HIBD represent an emerging public health threat and that research examining the health effects of HIBD is needed.^3^ The impact of alcohol on cardiovascular disease (CVD) and liver disease risk is partly predicted by changes in lipid profiles and liver function enzyme levels (LFTs),^5,6^ but previous studies either grouped individuals reporting HIBD using dichotomous binge thresholds or excluded them as outliers.

While the traditional binge thresholds have long been considered markers of development of risky alcohol use,^4^ these dichotomous variables neither distinguish between behavior of alcohol consumption just above the threshold and behavior far exceeding the threshold^7^ nor differentiate risk for anyone drinking above the threshold.^8^ Recent studies^4,9^ have shown that dose-dependent associations exist between health risk outcomes, such as illegal drug and tobacco use, risky sexual behavior, and injuries. However, the associations between HIBD and levels of both lipids and LFTs remain unknown.

Prompted by the revised 2018 Guideline on the Management of Blood Cholesterol recommendations^10^ and the increase in HIBD, we performed an ad hoc analysis of the NIAAA intramural clinical sample, which is enriched with individuals who engage in HIBD and contains more granular alcohol consumption data than generally available from population-based studies, to examine the associations between HIBD and levels of lipids and LFTs.

## Methods

### Study Population

This study followed the Strengthening the Reporting of Observational Studies in Epidemiology (STROBE) reporting guideline for cohort studies. The study was an ad hoc analysis of the NIAAA clinical cohort collected from March 3, 2005, through August 21, 2017. Participant data from this study came from 3 NIAAA protocols. Healthy volunteers and alcohol-dependent participants (diagnosed using the *Diagnostic and Statistical Manual of Mental Disorders, Fourth Edition*^11^) 18 years or older were recruited through local advertisements, word of mouth, or the NIAAA alcohol treatment program in Bethesda, Maryland. Women were excluded if they were breastfeeding, were pregnant, or intended to become pregnant. Written informed consent was obtained in accordance with the Declaration of Helsinki^12^ and the institutional review board of the NIAAA, which approved the study. Treatment-seeking participants were voluntarily admitted to the NIAAA inpatient unit in the National Institutes of Health Clinical Center. Following admission, treatment-seeking participants were detoxified from alcohol consumption and participated in the 28-day NIAAA alcohol treatment program, during which extensive clinical and physical examinations were performed. Healthy volunteers completed daylong screening assessments during which clinical characteristics and information regarding recent drinking history were collected.

### Alcohol Consumption Patterns

A standard drink was defined as any drink containing 14 g pure alcohol.^13^ The Timeline Followback (TLFB) questionnaire and interview uses a calendar and memory aids to improve recall of recent alcohol consumption behavior.^14^ We used the TLFB information related to alcohol consumption for the 90 days prior to screening or admission. Following the definitions by Hingson et al^4^ categorizing level I, II, and III as 1, 2, and 3 or more times drinking beyond the NIAAA binge threshold and the categorization by Patrick^7^ of levels II and III as high-intensity drinking (HIBD), we constructed a similar typology. However, instead of creating binge levels based on the reported maximum single-day alcohol consumption from the past 12 months, we took advantage of the TLFB’s day-level data and constructed our binge levels by dividing the total number of standard drinks consumed in the 90 days covered by the TLFB by the number of days with reported drinking, thus creating a drinks per drinking day (DPDD) variable. Our nonbinge group comprised men who reported consuming fewer than 5 DPDD on average over the 90 days prior to screening or admission and women who reported consuming fewer than 4 DPDD. Binge level I included men consuming between 5 and 9 DPDD and women consuming between 4 and 7 DPDD; level II included men consuming between 10 and 15 DPDD, and women consuming between 8 and 11 DPDD; and level III included men consuming more than 15 DPDD and women consuming 12 or more DPDD.

Using the reported day-level total alcohol consumption, we also constructed level I, II, and III binge frequencies, which we defined as the count of the number of days over the 90-day TFLB participants consumed at binge levels I, II, and III, as defined.

### Blood Collection

Blood and plasma samples were collected after 10 hours of fasting the morning after inpatient admission day, or during a screening visit, using standard methods. All blood samples were drawn at approximately 8 am after overnight bed rest and processed by the National Institutes of Health Clinical Center Department of Laboratory Medicine.

### Statistical Analysis

All statistical analyses were performed between December 3, 2018, and January 30, 2019, using R statistical software version 3.3.3 (R Project for Statistical Computing). We calculated descriptive statistics for the final analytical sample by binge levels using the compareGroups package in R. We note an additional 9 missing observations for low-density lipoprotein cholesterol (LDL-C), 1 for total cholesterol (TC), and 2 for aspartate aminotransferase (AST) were removed from the analyses. We used analysis of variance, Kruskall-Wallis, and χ^2^ or Fisher exact tests, depending on whether the variable was normal, continuous nonnormal, or categorical (distributions determined by compareGroups), respectively, to determine whether the distribution of the variables differed across and between binge levels.

To investigate the association between clinically high levels of lipids or LFT and HIBD, we defined clinically high levels of lipids based upon the values used in the Third Report of the Expert Panel on Detection, Evaluation, and Treatment of High Blood Cholesterol in Adults.^15^ We defined clinically high levels of γ-glutamyltransferase (GGT) based on the A.D.A.M. Medical Encyclopedia^16^ and levels of AST and alanine aminotransferase (ALT) based on the American College of Gastroenterology recommended guidelines.^17^ Using the definition, each biomarker was classified as a dichotomous outcome; we report the statistics across binge levels. We then used multivariable logistic regression models to isolate the associations between binge levels and clinically high levels of lipids and LFTs, adjusting for age, race, and body mass index (BMI; calculated as weight in kilograms divided by height in meters squared). We report the estimates as odds ratios (ORs) with 95% confidence intervals comparing the odds of presenting with a clinically high biomarker in the binge level compared with the odds in the non–binge level reference group.

We also used multivariable logistic regression to examine the association between and the frequency of consuming at each binge level (compared with the nonbinge reference) and clinically high levels of lipids and LFT biomarkers. The frequency was derived by counting the number of days (over the 90 days covered by the TLFB) participants reported consuming at each level; because the total number of days adds up to 90, we omitted the non–binge level count to avoid multicollinearity. Total number of drinks consumed (over the 90-day TLFB), age, sex, race, and BMI were included as covariates. Regression estimates are reported as ORs with 95% confidence intervals for the clinically high lipid or LFT level per additional day of consuming at the specified binge level. Goodness of fit was assessed by the Hosmer-Lemeshow test.

We hypothesized that levels of any of the 7 lipid or LFT biomarkers were differentially associated with levels of alcohol binge drinking (4 levels yielding 6 comparisons). Thus, we used a Bonferroni corrected threshold of *P* = .001 (nominal *P* = .05; 42 tests) to identify statistical significance for all analyses. All tests were 2-sided.

## Results

### Participants

A total of 2065 participants underwent protocol screening, and 1519 with available alcohol consumption, BMI, and lipid and LFT profiles were included in the final analyses. Descriptive statistics for the analytical cohort are presented (Table; eTable 1 in the Supplement). Mean (SD) age was 39.7 (12.1) years; mean (SD) BMI was 26.6 (5.1); 978 (64.4%) were male; 718 (47.3%) were white and 649 (42.7%) black/African American; and 578 (31.1%) consumed alcohol at the nonbinge level, 321 (21.2%) at level I, 239 (15.7%) at level II, and 381 (25.1%) at level III. In all, 99.5% of participants consuming at level III, 97% of participants at level II, 84.8% at level I, and 15.5% at the nonbinge level were diagnosed with alcohol dependence. Participants consuming at levels I, II, and III were older than those consuming at the nonbinge level. When lipid levels were tested, 9.8% of the participants presented with clinically high TC; 9.0%, HDL-C; 5.6%, LDL-C; and 8.8%, triglycerides (TRG). For LFTs, 35.9% had high levels of ALT; 28.9%, AST; and 56.4%, GGT (eTable 1 in the Supplement). Clinically high HDL-C significantly differed between nonbinge vs HIBD (levels II and III), and clinically high TRG and all LFTs significantly differed between nonbinge vs levels I and HIBD (levels II and III) consumption.

**Table.** Descriptive Statistics by Alcohol Binge Level

Characteristics	No. (%)					*P* Value, Overall
	All (N = 1519)	Nonbinge (n = 578)	Level I (n = 321)	Level II (n = 239)	Level III (n = 381)	
**Demographic and Clinical Characteristics**						
Age at admission, mean (SD), y	39.7 (12.1)	35.6 (12.5)	41.6 (12.1)	42.9 (10.8)	42.3 (10.4)	<.001^a^^,^^b^^,^^c^
Sex						<.001^a^^,^^b^^,^^c^
Male	978 (64.4)	314 (54.3)	216 (67.3)	173 (72.4)	275 (72.2)	
Female	541 (35.6)	264 (45.7)	105 (32.7)	66 (27.6)	106 (27.8)	
Race						
American Indian or Alaska Native	7 (0.5)	1 (0.2)	1 (0.3)	1 (0.4)	4 (1.0)	.25
Asian	50 (3.3)	35 (6.1)	10 (3.1)	1 (0.4)	4 (1.0)	<.001^b^^,^^c^
Black/African American	649 (42.7)	240 (41.5)	153 (47.7)	104 (43.5)	152 (39.9)	.18
Multiracial	39 (2.6)	18 (3.1)	6 (1.9)	9 (3.8)	6 (1.6)	.24
Unknown race	53 (3.5)	13 (2.2)	12 (3.7)	9 (3.8)	19 (5.0)	.15
White	718 (47.3)	269 (46.5)	139 (43.3)	115 (48.1)	195 (51.2)	.21
Body mass index, mean (SD)^d^	26.6 (5.1)	26.3 (4.9)	26.7 (5.3)	26.8 (5.1)	26.9 (5.0)	.20
**Alcohol-Related Characteristics**						
Alcohol dependence						
Current	923 (64.8)	82 (15.5)	251 (84.8)	225 (97.0)	365 (99.5)	<.001^a^^,^^b^^,^^c^^,^^e^^,^^f^
Past	298 (20.9)	56 (10.6)	78 (26.4)	61 (26.3)	103 (28.1)	<.001^a^^,^^b^^,^^c^
In prior 90 d, mean (SD)						
Drinks consumed, No.	642.0 (711.0)	76.5 (93.2)	433.0 (213.0)	821.0 (288.0)	1563.0 (723.0)	<.001^a^^,^^b^^,^^c^^,^^e^^,^^f^^,^^g^
Drinking days, No.	53.0 (32.1)	26.6 (25.9)	62.2 (25.6)	70.1 (21.6)	74.6 (21.3)	<.001^a^^,^^b^^,^^c^^,^^e^^,^^f^
Days with no drinks consumed, No.	37.0 (32.1)	63.4 (25.9)	27.8 (25.6)	19.8 (21.7)	15.3 (21.3)	<.001^a^^,^^b^^,^^c^^,^^e^^,^^f^
Days drinking, mean (SD), No.						
At nonbinge level	48.0 (35.7)	85.4 (8.5)	38.1 (25.7)	21.9 (21.8)	15.9 (21.4)	<.001^a^^,^^b^^,^^c^^,^^e^^,^^f^
At level I	12.0 (19.4)	4.3 (8.1)	38.1 (24.0)	11.6 (14.3)	2.1 (5.1)	<.001^a^^,^^b^^,^^e^^,^^f^^,^^g^
At level II	10.7 (19.6)	0.2 (1.0)	11.6 (14.2)	39.5 (27.8)	7.9 (13.3)	<.001^a^^,^^b^^,^^c^^,^^e^^,^^f^^,^^g^
At level III	19.2 (30.1)	0.1 (0.4)	2.2 (4.2)	17.1 (15.8)	64.1 (24.9)	<.001^b^^,^^c^^,^^e^^,^^f^^,^^g^

^a^ *P* < .001 denoted nonbinge vs level I.

^b^ *P* < .001 denoted nonbinge vs level II.

^c^ *P* < .001 denoted nonbinge vs level III.

^d^ Calculated as weight in kilograms divided by height in meters squared.

^e^ *P* < .001 denoted level I vs II.

^f^ *P* < .001 denoted level I vs III.

^g^ *P* < .001 denoted level II v III.

**Figure 1.**
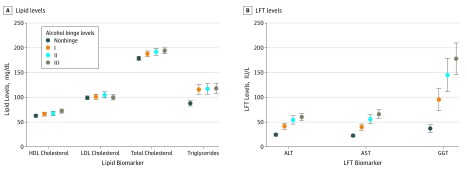
Means Levels of Lipid and Liver Function Test (LFT) Biomarkers by Alcohol Binge Levels Error bars indicate unadjusted 95% confidence intervals; ALT, alanine aminotransferase; AST, aspartate aminotransferase; GGT, γ-glutamyltransferase; HDL, high-density lipoprotein; and LDL, low-density lipoprotein.

Mean levels (with unadjusted 95% CIs) of lipids and LFTs are shown in Figure 1. Levels of TC, HDL-C, and TRG showed dose-dependent increases across binge levels (Figure 1A). Mean levels of TRG were highest for level III consumption (194.0 mg/dL; 95% CI, 189.4-198.6 mg/dL [to convert cholesterol to millimoles per liter, multiply by 0.0259]), with significant differences between nonbinge vs levels I, II, and III (*P* < .001). Mean levels of HDL-C were highest for level III consumption (72.1 mg/dL; 95% CI, 69.1-75.1 mg/dL), with significant differences between nonbinge vs HIBD level III and level I vs III consumption (*P* < .001). Mean levels of TRG were highest for level III (118.0 mg/dL; 95% CI, 109.0-127.0 mg/dL), with significant differences between nonbinge and levels I, II, and III consumption (*P* < .001). For LFTs ALT, AST, and GGT, mean levels showed similar dose-dependent increases across binge levels (Figure 1B), with mean levels of ALT (60.2 IU/L; 95% CI, 54.2-66.2 IU/L), AST (65.8 IU/L; 95% CI, 58.3-73.3 IU/L), and GGT (178.0 IU/L; 95% CI, 147.3-208.7 IU/L) highest for level III consumption. There were significant differences between nonbinge vs levels I, II, and III and between level I vs levels II and III consumption.

### Multivariable Analysis Examining the Association Between Binge Levels and Clinically High Levels of Lipids and LFTs

**Figure 2.**
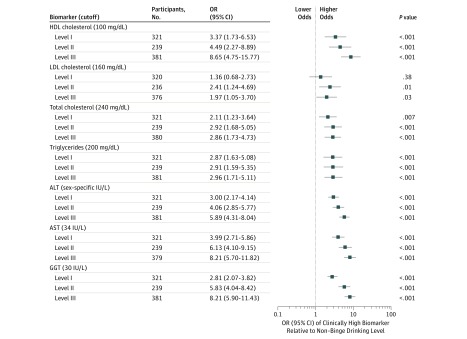
Odds Ratios (ORs) With 95% CIs for Clinically High Lipid and Liver Function Test Biomarkers by Alcohol Binge Levels Significance was set at adjusted Bonferroni threshold *P* < .001. Regression statistics are presented in eTable 2 in the Supplement. ALT indicates alanine aminotransferase; AST, aspartate aminotransferase; GGT, γ-glutamyltransferase; HDL, high-density lipoprotein; and LDL, low-density lipoprotein.

We found that alcohol consumption above the nonbinge level, especially at HIBD levels, was associated with increased odds of clinically high levels of HDL-C, TC, TRG, and LFTs (Figure 2; eTable 2 in the Supplement). Compared with reference nonbinge consumption, level I consumption was associated with more than triple the odds of clinically high HDL-C (OR, 3.37; 95% CI, 1.73-6.53; *P* < .001); level II consumption, more than 4 times the odds (OR, 4.49; 95% CI, 2.27-8.89; *P* < .001); and level III consumption, more than 8 times the odds (OR, 8.65; 95% CI, 4.75-15.77; *P* < .001). High-intensity binge drinking was associated with approximately triple the odds of clinically high TC for level II (OR, 2.91; 95% CI, 1.59-5.35; *P* < .001) and level III (OR, 2.96; 95% CI, 1.71-5.11; *P* < .001). The LFTs ALT, AST, and GGT showed similar binge-dependent responses: levels II and III consumption were associated with increased odds of clinically high LFTs by factors of 5 to 8 times the reference nonbinge level (eg, for GGT: level II OR, 5.83; 95% CI, 4.04-8.42 and level III OR, 8.21; 95% CI, 5.90-11.43; *P* < .001).

### Multivariable Analysis Examining the Associations Between Binge Drinking Frequency and Clinically High Lipid and LFT Levels

**Figure 3.**
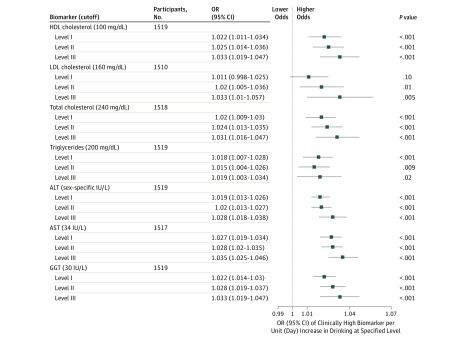
Odds Ratios (ORs) With 95% CIs for Clinically High Lipid and Liver Function Test Biomarkers by Alcohol Binge Levels Controlling for Total Alcohol Consumption Controlling for total alcohol consumption and other binge level frequencies. Significance was set at adjusted Bonferroni threshold *P* < .001. Regression statistics are presented in eTable 3 in the Supplement. ALT indicates alanine aminotransferase; AST, aspartate aminotransferase; GGT, γ-glutamyltransferase; HDL, high-density lipoprotein; and LDL, low-density lipoprotein. To convert cholesterol to millimoles per liter, multiply by 0.0259.

Simultaneously controlling for total number of alcoholic drinks consumed (total alcohol consumption) in the 90 days covered by the TLFB and drinking frequency at all other binge levels, we found increased frequency of HIBD (days consuming at levels II and III) was associated with increased odds of clinically high levels of HDL-C, TC, and all LFTs (per unit increase in days consuming at the respective binge level) (Figure 3; eTable 3 in the Supplement) (eg, for HDL-C: level II OR, 1.025; 95% CI, 1.014-1.036 and level III OR, 1.033; 95% CI, 1.019-1.047; for AST: level II OR, 1.028; 95% CI, 1.02-1.035 and level III OR, 1.035; 95% CI, 1.025-1.046; for GGT: level II OR, 1.028; 95% CI, 1.019-1.037 and level III OR, 1.033; 95% CI, 1.019-1.047; all *P* < .001). Notably, we did not find total alcohol consumption significantly associated with any clinically high lipid or LFT levels (eTable 3 in the Supplement).

## Discussion

Our data show HIBD was associated with increased levels of HDL-C, TC, TRG, ALT, AST, and GGT. Additionally, each binge level was associated with a stepwise increase in the odds of clinically high lipid and LFT levels. The association between alcohol consumption and cholesterol and TRG metabolism is well documented.^18^ Heavy alcohol consumption has been shown to contribute to hepatic steatosis^19^ and is associated with hypertriglyceridemia, possibly from increased chylomicron and very low-density lipoprotein levels.^20^ While the association between alcohol consumption and increased HDL-C levels has been previously described,^21^ our study is the first, to our knowledge, to report an association between HIBD and increased odds of extremely high HDL-C levels. Recent population-based studies have found a U-shaped association between HDL-C and all-cause mortality,^22,23^ with 1 study^22^ showing that men with HDL-C levels greater than 100 mg/dL had a 36% increased risk of all-cause mortality and women with HDL-C levels greater than 116 mg/dL had a 10% increased risk of all-cause mortality. While additional studies are needed to confirm such associations, our results suggest extremely high HDL-C may be an indirect marker of recent heavy alcohol consumption, which, given the association between alcohol consumption pattern and all-cause mortality risk,^24^ may explain, in part, the increase in all-cause mortality associated with extremely high HDL-C.^22^ Adverse cardiovascular effects from binge drinking are likely mediated by many physiological changes,^25^ and it is well known that an approximately 10% increase in LDL-C is associated with a 20% increase atherosclerosis risk.^26^ Given previous literature^25,27^ reporting an association of binge drinking, but not daily moderate alcohol consumption, with proatherogenic LDL-C (including Liu et al,^28^ who found that even when total alcohol consumption is the same, mice consuming alcohol in a binge pattern, compared with mice consuming daily moderate alcohol, had increased LDL-C levels with concomitant increase in atherosclerotic plaque development), we expected to find an association between HIBD and the odds of high levels of LDL-C. However, our results were not significant by the standards of this study. Further work is needed to elucidate the association between drinking patterns and LDL-C. Furthermore, we found each additional HIBD day was associated with clinically high circulating lipid and LFT levels, even after controlling for total alcohol consumption during the previous 90 days. While the association between HIBD and CVD is unknown, studies have found that people who drink in binges have higher rates of coronary heart disease^29^ and other CVD, including sudden cardiac death,^26^ compared with people reporting regular alcohol consumption. Since HIBD is common on weekends, sporting events, holidays, and special occasions,^7^ our results suggest HIBD, even occasionally, may have health implications.

The association between HIBD and LFTs is consistent with previous studies^30,31^ that have identified positive associations between alcohol and LFTs, including a recent mendelian randomization study^32^ suggesting a possible causal relationship. The significant increase in mean LFT levels from participants drinking at nonbinge levels and those drinking at each binge level suggests a possible hepatic response to HIBD. We found GGT showed the largest increases associated with HIBD, which suggests that GGT may be most sensitive to HIBD. γ-Glutamyltransferase maintains concentrations of glutathione and is important for defense against oxidant stress,^33^ and increased levels indicate antioxidant deficiency^33^ and reactive oxygen species production.^34^ Notably, several population-based studies have shown GGT to be associated CVD mortality.^33,35,36^ While evidence linking serum transaminase levels to CVD risk is not as strong as what is observed for GGT,^6^ clinically high ALT levels have been linked to stroke risk^37^ and increased CVD mortality.^38^ Furthermore, circulating levels of hepatic enzymes are considered good indicators of general health and long-term survival,^39^ and unexplained elevations in LFTs constitute a common cause of referral to liver clinics.^40^ Our findings suggest clinical interpretation of LFT findings may benefit from understanding recent drinking history, if deemed appropriate.

Our findings also suggest that drinking reduction interventions and strategies aimed at reducing HIBD to lower drinking levels may be associated with improved health outcomes. Compared with traditional treatment strategies aimed at total abstinence, more recent intervention strategies have widened treatment goals to include reduced alcohol intake.^21^ In our sample, for example, total abstinence may not be an achievable short-term goal for individuals in level III; however, our findings show that reducing consumption to level II or level I may be associated with improved biomarker levels, which could have a health impact. Studies have shown the benefit of alcohol reduction on lipid and LFT levels,^41,42^ slower progression in alcohol-related liver fibrosis,^43^ and improved psychological well-being.^44^ Therefore, further studies are needed to examine the health effects of reducing HIBD to lower drinking levels.

### Limitations

This study has limitations. First, the study was cross-sectional, which limits the assessment of causal relationships. We are also unable to draw any conclusions regarding the contribution of HIBD to CVD and liver disease. Second, because data came from volunteers participating in NIAAA screening protocols or seeking treatment for alcohol dependence, selection bias may be present, which may affect the results. Relatedly, the study cohort is not a random sample and results may not be representative of the general population. Third, the participants with alcohol dependence composing a majority of the HIBD groups may not be representative of individuals who engage in binge drinking in the population. However, Hingson et al^4^ found alcohol dependence to be the most robust predictor of HIBD in a nationally representative sample, and a positive response to the question, “On any single occasion during the past 3 months, have you had more than 5 drinks containing alcohol?” has been shown to accurately detect alcohol abuse and dependence,^45^ suggesting an association between HIBD and alcohol dependence in the general population. Similarly, the HIBD groups were older than the nonbinge group, and given that HIBD is prevalent among young adults,^3^ further studies are necessary to better understand the long-term effects of HIBD on circulating lipid levels and liver function in young adults. In addition, self-reported alcohol consumption data are prone to social desirability and recall biases,^46^ which could affect binge group composition. Fourth, there is the possibility of measurement bias due to admission procedure at the NIAAA inpatient treatment program: blood measurements for treatment-seeking participants were taken the day after admission, while they were obtained during the outpatient screening visit for the healthy volunteers. Fifth, while we adjusted for age, race, and BMI in the analyses, other residual confounders, including nutrition, diet, smoking status, and physical activity, may bias the multivariable estimates. Additionally, some studies^32,47^ have shown an interaction between BMI and alcohol consumption with LFTs; however, in this study, BMI did not significantly differ between alcohol binge levels, and we controlled for BMI in the multivariable analyses.

## Conclusions

High-intensity binge drinking was associated with dose-dependent increases in lipid and LFT levels. Multivariable analysis controlling for total alcohol consumption suggested that an additional day of level II or level III drinking was associated with increased odds of clinically high levels of HDL-C, TC, ALT, AST, and GGT. Our results provide support for the notion that the recent alarming trend in HIBD may have negative health consequences, as measured by lipid and LFT biomarkers. Further research is warranted to assess additional clinical implications. Similarly, in accordance with harm reduction approaches to alcohol consumption, our findings suggest interventions targeted at reducing HIBD may be important in improving health outcomes.

## Notes

Supplement. **eTable 1.** Clinically High Lipid and LFT Biomarkers by Alcohol Binge Level **eTable 2.** Logistic Regression Results for Alcohol Binge Levels on Clinically High Lipid and LFT Levels **eTable 3.** Logistic Regression Results for Alcohol Binge Intake Frequency on Clinically High Lipid and LFT Levels
