# Supplementary material for: Association of High-Intensity Binge Drinking With Lipid and Liver Function Enzyme Levels
Source: JAMA Netw Open. 2019 Jun 14;2(6):e195844. doi: 10.1001/jamanetworkopen.2019.5844 (PMC6575145; doi:10.1001/jamanetworkopen.2019.5844)
Supplement: Supplement. — eTable 1. Clinically High Lipid and LFT Biomarkers by Alcohol Binge Level eTable 2. Logistic Regression Results for Alcohol Binge Levels on Clinically High Lipid and LFT Levels eTable 3. Logistic Regression Results for Alcohol Binge Intake Frequency on Clinically High Lipid and LFT Levels [file jamanetwopen-2-e195844-s001.pdf]

Supplementary Online Content

Rosoff DB, Charlet K, Jung J, et al. Association of high-intensity binge drinking with lipid and liver function enzyme levels. *JAMA Netw Open*. 2019;2(6):e195844. doi:10.1001/jamanetworkopen.2019.5844

eTable 1. Clinically High Lipid and LFT Biomarkers by Alcohol Binge Level
eTable 2. Logistic Regression Results for Alcohol Binge Levels on Clinically High Lipid and LFT Levels
eTable 3. Logistic Regression Results for Alcohol Binge Intake Frequency on Clinically High Lipid and LFT Levels

This supplementary material has been provided by the authors to give readers additional information about their work.

© 2019 Rosoff DB et al. *JAMA Network Open.*

eTable 1. Clinically High Lipid and LFT Biomarkers by Alcohol Binge Level

Biomarker	All N=1519	Non-Binge N=578	Level I N=321	Level II N=239	Level III N=381	*P*-overall
**Lipid Biomarkers (mg/dL)**						
Total cholesterol (1 mo)						<.001[a-c]
< 240	1369 (90.2%)	552 (95.5%)	287 (89.4%)	204 (85.4%)	326 (85.8%)	
≥ 240	149 (9.82%)	26 (4.50%)	34 (10.6%)	35 (14.6%)	54 (14.2%)	
HDL cholesterol						<.001[a-c,e,f]
< 100	1383 (91.0%)	563 (97.4%)	294 (91.6%)	214 (89.5%)	312 (81.9%)	
≥ 100	136 (8.95%)	15 (2.60%)	27 (8.41%)	25 (10.5%)	69 (18.1%)	
LDL cholesterol (9 mo)						.002 [b,c]
< 160	1425 (94.4%)	560 (96.9%)	303 (94.7%)	214 (90.7%)	348 (92.6%)	
≥ 160	85 (5.63%)	18 (3.11%)	17 (5.31%)	22 (9.32%)	28 (7.45%)	
Triglycerides						<.001[a-c]
< 200	1386 (91.2%)	556 (96.2%)	286 (89.1%)	211 (88.3%)	333 (87.4%)	
≥ 200	133 (8.76%)	22 (3.81%)	35 (10.9%)	28 (11.7%)	48 (12.6%)	
**LFT Biomarkers (IU/L)**						
Alanine Aminotransferase (ALT)						<.001[a-c,e]
< 25 females; < 40 males	974 (64.1%)	477 (82.5%)	198 (61.7%)	129 (54.0%)	170 (44.6%)	
≥ 25 females; ≥ 40 males	545 (35.9%)	101 (17.5%)	123 (38.3%)	110 (46.0%)	211 (55.4%)	
Aspartate Aminotransferase (AST) (2 mo)						<.001[a-e]
< 34	1078 (71.1%)	527 (91.2%)	221 (68.8%)	138 (57.7%)	192 (50.7%)	
≥ 34	439 (28.9%)	51 (8.82%)	100 (31.2%)	101 (42.3%)	187 (49.3%)	
Gamma-glutamyltransferase (GGT)						<.001[a-e]
< 30	662 (43.6%)	405 (70.1%)	128 (39.9%)	57 (23.8%)	72 (18.9%)	
≥ 30	857 (56.4%)	173 (29.9%)	193 (60.1%)	182 (76.2%)	309 (81.1%)	

Notes: Number of cases of clinically high levels (%) reported. Participants with missing observations (mo) were excluded from multivariable analyses. Abbreviations: HDL: high-density lipoprotein; LDL: low-density lipoprotein; mg/dL: milligrams per deciliter; IU/L: International Unit per Liter; LFT: liver function tests. Pairwise comparisons significant at Bonferroni adjusted threshold *P* <.001 denoted: "a" non-binge v. level I; "b" non-binge v. level II; "c" non-binge v. level III; "d" level 1 v. II; "e" level I v. III; "f" level II v III.

© 2019 Rosoff DB et al. *JAMA Network Open.*

**eTable 2. Logistic Regression Results for Alcohol Binge Levels on Clinically High Lipid and LFT Levels**														
										**Adjustment: Bonferroni method for 42 comparisons**				
**Predictors**	**Estimate**	**SE**	**Z**	**Pr(>|z|)**	**LCI**	**UCI**	**OR**	**OR LCI**	**OR UCI**	**LCI**	**UCI**	**OR LCI**	**OR LCI**	**P value**
**Outcome: Clinically High HDL-C**														
(Intercept)	-1.589	0.665	-2.388	1.70E-02	-2.893	-0.285	0.204	0.055	0.752	-3.745	0.568	0.024	1.764	7.12E-01
Level I alcohol consumption	1.214	0.338	3.588	3.33E-04	0.551	1.876	3.365	1.734	6.531	0.117	2.310	1.124	10.073	1.00E+00
Level II alcohol consumption	1.502	0.348	4.315	1.60E-05	0.820	2.184	4.491	2.270	8.885	0.374	2.630	1.453	13.878	6.72E-04
Level III alcohol consumption	2.158	0.306	7.047	1.83E-12	1.558	2.758	8.652	4.747	15.767	1.165	3.150	3.207	23.340	7.69E-11
Age	0.018	0.009	2.132	3.30E-02	0.001	0.035	1.018	1.001	1.036	-0.010	0.046	0.991	1.047	1.00E+00
Race: Black/ African-American	0.038	0.195	0.196	8.44E-01	-0.344	0.420	1.039	0.709	1.522	-0.593	0.670	0.553	1.954	1.00E+00
Race: Asian	0.103	0.640	0.160	8.72E-01	-1.151	1.356	1.108	0.316	3.882	-1.971	2.176	0.139	8.810	1.00E+00
Race: American Indian/ Native American	-0.242	1.151	-0.210	8.34E-01	-2.498	2.014	0.785	0.082	7.491	-3.972	3.488	0.019	32.726	1.00E+00
Multiracial	-0.347	0.751	-0.462	6.44E-01	-1.819	1.125	0.707	0.162	3.081	-2.782	2.088	0.062	8.065	1.00E+00
Gender: Male	-0.428	0.197	-2.172	2.98E-02	-0.814	-0.042	0.652	0.443	0.959	-1.067	0.211	0.344	1.234	1.00E+00
BMI	-0.099	0.022	-4.440	9.01E-06	-0.142	-0.055	0.906	0.868	0.946	-0.171	-0.027	0.843	0.974	3.78E-04
Null Deviance (df)	915.82 (1518)													
Residual Deviance (df)	812.51 (1508)													
Observations deleted due to missingness	0													
AIC	834.51													
Hoslem-Lemeshow Goodness of Fit: P-value	> 0.99													
										**Adjustment: Bonferroni method for 42 comparisons**				
**Outcome: Clinically High LDL-C**	**Estimate**	**SE**	**Z**	**Pr(>|z|)**	**LCI**	**UCI**	**OR**	**OR LCI**	**OR UCI**	**LCI**	**UCI**	**OR LCI**	**OR LCI**	**P value**
(Intercept)	-7.285	0.803	-9.071	2.00E-16	-8.859	-5.711	0.001	0.000	0.003	-9.888	-4.682	0.000	0.009	8.40E-15
Level I alcohol consumption	0.309	0.355	0.872	3.83E-01	-0.386	1.004	1.362	0.680	2.730	-0.840	1.459	0.432	4.300	1.00E+00
Level II alcohol consumption	0.880	0.340	2.593	9.52E-03	0.215	1.546	2.412	1.240	4.693	-0.220	1.981	0.802	7.250	4.00E-01
Level III alcohol consumption	0.678	0.321	2.110	3.48E-02	0.048	1.307	1.969	1.049	3.696	-0.363	1.719	0.695	5.578	1.00E+00
Age	0.052	0.011	4.870	1.12E-06	0.031	0.073	1.053	1.031	1.075	0.017	0.086	1.017	1.090	4.70E-05
Race: Black/ African-American	-0.167	0.238	-0.702	4.83E-01	-0.633	0.299	0.846	0.531	1.349	-0.938	0.604	0.391	1.829	1.00E+00
Race: Asian	-0.250	1.041	-0.241	8.10E-01	-2.290	1.789	0.778	0.101	5.986	-3.623	3.123	0.027	22.706	1.00E+00
Race: American Indian/ Native American	0.053	1.113	0.047	9.62E-01	-2.129	2.235	1.054	0.119	9.345	-3.556	3.661	0.029	38.905	1.00E+00
Multiracial	-0.898	1.036	-0.867	3.86E-01	-2.928	1.132	0.407	0.053	3.100	-4.255	2.458	0.014	11.686	1.00E+00
Gender: Male	-0.053	0.246	-0.214	8.30E-01	-0.534	0.429	0.949	0.586	1.535	-0.848	0.743	0.428	2.103	1.00E+00
BMI	0.069	0.020	3.469	5.22E-04	0.030	0.108	1.071	1.030	1.114	0.005	0.133	1.005	1.143	2.19E-02
Null Deviance (df)	654.25 (1509)													
Residual Deviance (df)	597.61 (1499)													
Observations deleted due to missingness	9													
AIC	619.61													
Hoslem-Lemeshow Goodness of Fit: P-value	> 0.99													

© 2019 Rosoff DB et al. *JAMA Network Open.*

										Adjustment: Bonferroni method for 42 comparisons				
**Outcome: Clinically HighTotal Cholesterol**	**Estimate**	**SE**	**Z**	**Pr(>|z|)**	**LCI**	**UCI**	**OR**	**OR LCI**	**OR UCI**	**LCI**	**UCI**	**OR LCI**	**OR LCI**	**P value**
(Intercept)	-5.086	0.597	-8.520	2.00E-16	-6.256	-3.916	0.006	0.002	0.020	-7.020	-3.151	0.001	0.043	8.40E-15
Level I alcohol consumption	0.747	0.277	2.694	7.07E-03	0.203	1.291	2.111	1.226	3.635	-0.152	1.646	0.859	5.186	2.97E-01
Level II alcohol consumption	1.070	0.280	3.816	1.36E-04	0.521	1.620	2.916	1.683	5.054	0.161	1.979	1.175	7.239	5.71E-03
Level III alcohol consumption	1.052	0.257	4.098	4.17E-05	0.549	1.555	2.863	1.731	4.734	0.220	1.884	1.246	6.578	1.75E-03
Age	0.040	0.008	5.032	4.86E-07	0.025	0.056	1.041	1.025	1.058	0.014	0.066	1.014	1.068	2.04E-05
Race: Black/ African-American	-0.458	0.188	-2.437	1.48E-02	-0.826	-0.090	0.633	0.438	0.914	-1.067	0.151	0.344	1.163	6.23E-01
Race: Asian	-0.406	0.746	-0.544	5.87E-01	-1.868	1.057	0.667	0.154	2.878	-2.824	2.013	0.059	7.486	1.00E+00
Race: American Indian/ Native American	-0.641	1.099	-0.583	5.60E-01	-2.794	1.513	0.527	0.061	4.539	-4.202	2.920	0.015	18.546	1.00E+00
Multiracial	-0.868	0.746	-1.163	2.45E-01	-2.330	0.595	0.420	0.097	1.813	-3.286	1.551	0.037	4.716	1.00E+00
Gender: Male	-0.232	0.187	-1.240	2.15E-01	-0.597	0.134	0.793	0.550	1.144	-0.837	0.374	0.433	1.453	1.00E+00
BMI	0.030	0.017	1.829	6.75E-02	-0.002	0.063	1.031	0.998	1.065	-0.024	0.084	0.977	1.088	1.00E+00
Null Deviance (df)	974.59 (1517)													
Residual Deviance (df)	899.68 (1507)													
Observations deleted due to missingness	2													
AIC	921.68													
Hoslem-Lemeshow Goodness of Fit: P-value	> 0.99													
										**Adjustment: Bonferroni method for 42 comparisons**				
**Outcome: Clinically High Triglycerides**	**Estimate**	**SE**	**Z**	**Pr(>|z|)**	**LCI**	**UCI**	**OR**	**OR LCI**	**OR UCI**	**LCI**	**UCI**	**OR LCI**	**OR LCI**	**P value**
(Intercept)	-6.426	0.642	-10.017	2.00E-16	-7.684	-5.169	0.002	0.000	0.006	-8.505	-4.347	0.000	0.013	8.40E-15
Level I alcohol consumption	1.055	0.291	3.629	2.84E-04	0.485	1.625	2.872	1.625	5.078	0.113	1.997	1.119	7.370	1.19E-02
Level II alcohol consumption	1.069	0.310	3.447	5.67E-04	0.461	1.677	2.913	1.586	5.350	0.064	2.074	1.066	7.960	2.38E-02
Level III alcohol consumption	1.085	0.279	3.891	9.97E-05	0.538	1.631	2.959	1.713	5.111	0.181	1.989	1.199	7.305	4.19E-03
Age	0.008	0.008	0.954	3.40E-01	-0.008	0.024	1.008	0.992	1.024	-0.019	0.035	0.981	1.035	1.00E+00
Race: Black/ African-American	-0.794	0.208	-3.824	1.31E-04	-1.200	-0.387	0.452	0.301	0.679	-1.466	-0.121	0.231	0.886	5.50E-03
Race: Asian	0.217	0.564	0.386	7.00E-01	-0.888	1.323	1.243	0.412	3.754	-1.610	2.045	0.200	7.733	1.00E+00
Race: American Indian/ Native American	0.068	1.122	0.060	9.52E-01	-2.132	2.268	1.070	0.119	9.657	-3.570	3.706	0.028	40.679	1.00E+00
Multiracial	-0.877	0.750	-1.171	2.42E-01	-2.347	0.592	0.416	0.096	1.807	-3.307	1.552	0.037	4.721	1.00E+00
Gender: Male	0.642	0.225	2.852	4.34E-03	0.201	1.084	1.901	1.222	2.955	-0.088	1.372	0.916	3.943	1.82E-01
BMI	0.102	0.017	6.060	1.36E-09	0.069	0.135	1.108	1.072	1.145	0.048	0.157	1.049	1.170	5.71E-08
Null Deviance (df)	901.83 (1518)													
Residual Deviance (df)	815 (1508)													
Observations deleted due to missingness	0													
AIC	837.00													
Hoslem-Lemeshow Goodness of Fit: P-value	> 0.99													
										**Adjustment: Bonferroni method for 42 comparisons**				
**Outcome: Clinically High GGT**	**Estimate**	**SE**	**Z**	**Pr(>|z|)**	**LCI**	**UCI**	**OR**	**OR LCI**	**OR UCI**	**LCI**	**UCI**	**OR LCI**	**OR LCI**	**P value**
(Intercept)	-4.269	0.400	-10.681	2.00E-16	-5.052	-3.485	0.014	0.006	0.031	-5.564	-2.973	0.004	0.051	8.40E-15

© 2019 Rosoff DB et al. *JAMA Network Open.*

Level I alcohol consumption	1.033	0.156	6.614	3.74E-11	0.727	1.339	2.809	2.068	3.815	0.527	1.539	1.693	4.660	1.57E-09
Level II alcohol consumption	1.763	0.187	9.404	2.00E-16	1.396	2.131	5.830	4.038	8.420	1.155	2.371	3.175	10.705	8.40E-15
Level III alcohol consumption	2.106	0.168	12.498	2.00E-16	1.775	2.436	8.212	5.903	11.425	1.560	2.652	4.757	14.178	8.40E-15
Age	0.038	0.005	7.156	8.29E-13	0.027	0.048	1.038	1.028	1.049	0.021	0.055	1.021	1.056	3.48E-11
Race: Black/ African-American	0.278	0.128	2.166	3.03E-02	0.026	0.530	1.321	1.027	1.698	-0.138	0.694	0.871	2.002	1.00E+00
Race: Asian	0.065	0.358	0.183	8.55E-01	-0.636	0.767	1.068	0.529	2.153	-1.095	1.226	0.335	3.406	1.00E+00
Race: American Indian/ Native American	-0.827	0.822	-1.007	3.14E-01	-2.439	0.784	0.437	0.087	2.190	-3.492	1.837	0.030	6.278	1.00E+00
Multiracial	-0.350	0.396	-0.886	3.76E-01	-1.126	0.425	0.704	0.324	1.530	-1.633	0.932	0.195	2.539	1.00E+00
Gender: Male	0.910	0.127	7.141	9.29E-13	0.660	1.160	2.484	1.935	3.188	0.497	1.323	1.644	3.754	3.90E-11
BMI	0.052	0.012	4.198	2.69E-05	0.028	0.076	1.053	1.028	1.079	0.012	0.092	1.012	1.096	1.13E-03
Null Deviance (df)	2080.7 (1518)													
Residual Deviance (df)	1622.8 (1508)													
Observations deleted due to missingness	0													
AIC	1644.80													
**Hoslem-Lemeshow Goodness of Fit: P-value**	> 0.99													
										**Adjustment: Bonferroni method for 42 comparisons**				
**Outcome: Clinically High ALT**	**Estimate**	**SE**	**Z**	**Pr(>|z|)**	**LCI**	**UCI**	**OR**	**OR LCI**	**OR UCI**	**LCI**	**UCI**	**OR LCI**	**OR LCI**	**P value**
(Intercept)	-2.334	0.362	-6.447	1.14E-10	-3.044	-1.625	0.097	0.048	0.197	-3.508	-1.161	0.030	0.313	4.79E-09
Level I alcohol consumption	1.097	0.165	6.639	3.16E-11	0.773	1.421	2.995	2.167	4.141	0.561	1.633	1.753	5.118	1.33E-09
Level II alcohol consumption	1.400	0.179	7.802	6.11E-15	1.049	1.752	4.057	2.853	5.767	0.819	1.982	2.267	7.258	2.57E-13
Level III alcohol consumption	1.773	0.159	11.127	2.00E-16	1.460	2.085	5.886	4.308	8.044	1.256	2.289	3.512	9.865	8.40E-15
Age	0.015	0.005	2.953	3.15E-03	0.005	0.025	1.015	1.005	1.025	-0.001	0.031	0.999	1.032	1.32E-01
Race: Black/ African-American	-0.754	0.123	-6.143	8.12E-10	-0.995	-0.514	0.470	0.370	0.598	-1.152	-0.356	0.316	0.700	3.41E-08
Race: Asian	-0.038	0.345	-0.111	9.12E-01	-0.715	0.638	0.962	0.489	1.893	-1.157	1.080	0.315	2.945	1.00E+00
Race: American Indian/ Native American	-1.526	0.864	-1.765	7.76E-02	-3.220	0.168	0.218	0.040	1.183	-4.327	1.276	0.013	3.581	1.00E+00
Multiracial	-0.473	0.378	-1.252	2.11E-01	-1.215	0.268	0.623	0.297	1.307	-1.699	0.753	0.183	2.122	1.00E+00
Gender: Male	-0.367	0.124	-2.964	3.03E-03	-0.609	-0.124	0.693	0.544	0.883	-0.768	0.034	0.464	1.035	1.27E-01
BMI	0.028	0.011	2.422	1.55E-02	0.005	0.050	1.028	1.005	1.051	-0.009	0.065	0.991	1.067	6.49E-01
Null Deviance (df)	1983 (1518)													
Residual Deviance (df)	1756.1 (1508)													
Observations deleted due to missingness	0													
AIC	1778.10													
Hoslem-Lemeshow Goodness of Fit: P-value	> 0.99													
										**Adjustment: Bonferroni method for 42 comparisons**				
**Outcome: Clinically High AST**	**Estimate**	**SE**	**Z**	**Pr(>|z|)**	**LCI**	**UCI**	**OR**	**OR LCI**	**OR UCI**	**LCI**	**UCI**	**OR LCI**	**OR LCI**	**P value**
(Intercept)	-3.037	0.430	-7.068	1.57E-12	-3.880	-2.195	0.048	0.021	0.111	-4.430	-1.645	0.012	0.193	6.59E-11
Level I alcohol consumption	1.383	0.197	7.022	2.19E-12	0.997	1.768	3.985	2.709	5.862	0.744	2.021	2.105	7.543	9.20E-11
Level II alcohol consumption	1.812	0.205	8.840	2.00E-16	1.411	2.214	6.125	4.098	9.154	1.148	2.477	3.151	11.903	8.40E-15
Level III alcohol consumption	2.105	0.186	11.331	2.00E-16	1.741	2.470	8.210	5.704	11.817	1.503	2.708	4.496	14.993	8.40E-15
Age	0.037	0.006	6.549	5.80E-11	0.026	0.048	1.038	1.026	1.049	0.019	0.055	1.019	1.057	2.44E-09
Race: Black/ African-American	-0.780	0.134	-5.838	5.27E-09	-1.042	-0.518	0.458	0.353	0.596	-1.213	-0.347	0.297	0.707	2.21E-07

© 2019 Rosoff DB et al. *JAMA Network Open.*

Race: Asian	-0.359	0.463	-0.775	4.38E-01	-1.266	0.548	0.698	0.282	1.730	-1.859	1.141	0.156	3.130	1.00E+00
Race: American Indian/ Native American	-1.144	0.874	-1.310	1.90E-01	-2.857	0.568	0.318	0.057	1.765	-3.976	1.688	0.019	5.407	1.00E+00
Multiracial	-0.867	0.460	-1.886	5.93E-02	-1.768	0.034	0.420	0.171	1.035	-2.357	0.623	0.095	1.865	1.00E+00
Gender: Male	0.513	0.141	3.633	2.81E-04	0.236	0.790	1.670	1.266	2.203	0.055	0.971	1.057	2.639	1.18E-02
BMI	-0.026	0.013	-1.957	5.03E-02	-0.052	0.000	0.974	0.950	1.000	-0.069	0.017	0.934	1.017	1.00E+00
Null Deviance (df)	1825.9 (1517)													
Residual Deviance (df)	1503 (1507)													
Observations deleted due to missingness	2													
AIC	1525.00													
Hoslem-Lemeshow Goodness of Fit: P-value	> 0.99													
**NOTES:**														
Predictor	Type	Sex specific	Definition:											
**Outcomes:**	**Type**	**Sex-specific**	**Definition**											
Clinically High HDL cholesterol	Dummy	no	1 if HDL-C ≥ 100 mg/dL, 0 otherwise											
Clinically High LDL cholesterol	Dummy	no	1 if LDL-C ≥ 160 mg/dL, 0 otherwise											
Clinically High Total Cholesterol	Dummy	no	1 if TC ≥ 240 mg/dL, 0 otherwise											
Clinically High Triglycerides	Dummy	no	1 if Triglycerides ≥ 200 mg/dL, 0 otherwise											
Clinically High ALT	Dummy	yes	Females: 1 if ALT ≥ 25 IU/L, 0 otherwise; Males: 1 if ALT ≥ 40 IU/L, 0 otherwise											
Clinically High AST	Dummy	no	1 if LDL-C ≥ 160 mg/dL, 0 otherwise											
Clinically High GGT	Dummy	no	1 if TC ≥ 240 mg/dL, 0 otherwise											
**Predictor:**	**Type**	**Sex-specific**	**Definition**											
Reference level: Non-binge alcohol consumption	Dummy	yes	Females: 1 if consume < 4 standard drinks on average over days drinking, 0 otherwise; Males: 1 if consume < 5 standard drinks on average over days drinking, 0 otherwise											
Level I alcohol consumption	Dummy	yes	Females: 1 if consume [4,8) standard drinks on average over days drinking, 0 otherwise; Males: 1 if consume [5,10) standard drinks on average over days drinking, 0 otherwise											
Level II alcohol consumption	Dummy	yes	Females: 1 if consume [8,12) standard drinks on average over days drinking, 0 otherwise; Males: 1 if consume [10,15) standard drinks on average over days drinking, 0 otherwise											
Level III alcohol consumption	Dummy	yes	Females: 1 if consume ≥12 standard drinks on average over days drinking, 0 otherwise; Males: 1 if consume ≥15 standard drinks on average over days drinking, 0 otherwise											
**Covariates:**	**Type**	**Sex-specific**	**Definition**											
Age	Continuous	no	Age at protocol screening or admission as in-patient if admitted											
Gender	Dummy	na	1 if male, 0 if female											
BMI	Continuous	no	body mass index											
Race	Dummy	no	1 if specified race, 0 otherwise											
**Abbreviations**														

© 2019 Rosoff DB et al. *JAMA Network Open.*

CI Adjustment:	Bonferroni method for 21 tests with qnorm(1-(.05/2*21))=3.038
P value adjustment:	Bonferroni method for 21 tests
SE	standard error of estimate
OR	odds ratio (since estimate is ln(OR), OR=EXP(estimate))
LCI	lower confidence interval bound
UCI	upper confidence interval bound
Hoslem-Lemeshow test adjusted P-value	Bonferroni method for 14 tests (7 tests, 2 multivariable analyses): Good fit if *P* > .004 Bonferroni adjusted threshold)

© 2019 Rosoff DB et al. *JAMA Network Open.*

eTable 3. Logistic Regression Results for Alcohol Binge Intake Frequency on Clinically High Lipid and LFT Levels														
										**Adjustment: Bonferroni method for 42 comparisons**				
**Predictors:**	**Estimate**	**SE**	**Z**	**Pr(>|z|)**	**LCI**	**UCI**	**OR**	**OR LCI**	**OR UCI**	**P value**	**LCI**	**UCI**	**OR LCI**	**OR LCI**
**Outcome: Clinically High HDL**														
(Intercept)	-1.359	0.652	-2.086	3.70E-02	-2.637	-0.082	0.257	0.072	0.921	1.00E+00	-3.472	0.753	0.031	2.123
Count of days drinking at level I	0.022	0.006	3.92	8.84E-05	0.011	0.033	1.022	1.011	1.034	3.71E-03	0.004	0.040	1.004	1.041
Count of days drinking at level II	0.024	0.005	4.497	6.89E-06	0.014	0.035	1.025	1.014	1.036	2.89E-04	0.007	0.042	1.007	1.043
Count of days drinking at level III	0.032	0.007	4.634	3.59E-06	0.019	0.046	1.033	1.019	1.047	1.51E-04	0.010	0.055	1.010	1.056
Total Drinks	0.000	0.000	-0.612	5.41E-01	-0.001	0.000	1.000	0.999	1.000	1.00E+00	-0.001	0.001	0.999	1.001
Age at admission	0.012	0.009	1.318	1.87E-01	-0.006	0.029	1.012	0.994	1.030	1.00E+00	-0.017	0.041	0.983	1.042
Race: Black/ African-American	-0.058	0.197	-0.294	7.69E-01	-0.444	0.328	0.944	0.641	1.389	1.00E+00	-0.696	0.581	0.498	1.787
Race: Asian	0.069	0.647	0.107	9.15E-01	-1.200	1.338	1.072	0.301	3.811	1.00E+00	-2.029	2.167	0.131	8.735
Race: American Indian/ Native American	0.358	1.133	0.316	7.52E-01	-1.863	2.579	1.431	0.155	13.188	1.00E+00	-3.314	4.031	0.036	56.318
Multiracial	-0.333	0.756	-0.44	6.60E-01	-1.815	1.150	0.717	0.163	3.157	1.00E+00	-2.783	2.118	0.062	8.317
Gender: Male	-0.470	0.206	-2.279	2.26E-02	-0.874	-0.066	0.625	0.417	0.936	9.49E-01	-1.138	0.198	0.321	1.219
BMI	-0.097	0.022	-4.336	1.45E-05	-0.140	-0.053	0.908	0.869	0.948	6.09E-04	-0.169	-0.024	0.845	0.976
Null Deviance (df)	915.82 (1518)													
Residual Deviance (df)	796.01 (1507)													
Observations deleted due to missingness	0													
AIC	820.01													
Hoslem-Lemeshow Goodness of Fit: P-value	> 0.99													
										**Adjustment: Bonferroni method for 42 comparisons**				
**Outcome: Clinically High LDL**	**Estimate**	**SE**	**Z**	**Pr(>|z|)**	**LCI**	**UCI**	**OR**	**OR LCI**	**OR UCI**	**P value**	**LCI**	**UCI**	**OR LCI**	**OR LCI**
(Intercept)	-7.220	0.788	-9.163	2.00E-16	-8.764	-5.675	0.001	0.000	0.003	8.40E-15	-9.773	-4.666	0.000	0.009
Count of days drinking at level I	0.011	0.007	1.63	1.03E-01	-0.002	0.025	1.011	0.998	1.025	1.00E+00	-0.011	0.034	0.989	1.034
Count of days drinking at level II	0.020	0.008	2.582	9.84E-03	0.005	0.035	1.020	1.005	1.036	4.13E-01	-0.005	0.045	0.995	1.046
Count of days drinking at level III	0.032	0.012	2.787	5.33E-03	0.010	0.055	1.033	1.010	1.057	2.24E-01	-0.005	0.070	0.995	1.073
Total Drinks	-0.001	0.001	-2.019	4.35E-02	-0.002	0.000	0.999	0.998	1.000	1.00E+00	-0.003	0.001	0.997	1.001
Age at admission	0.048	0.011	4.525	6.05E-06	0.027	0.069	1.050	1.028	1.072	2.54E-04	0.014	0.083	1.014	1.087
Race: Black/ African-American	-0.168	0.239	-0.703	4.82E-01	-0.637	0.301	0.845	0.529	1.351	1.00E+00	-0.943	0.607	0.389	1.835
Race: Asian	-0.321	1.039	-0.309	7.57E-01	-2.358	1.715	0.725	0.095	5.558	1.00E+00	-3.689	3.047	0.025	21.043
Race: American Indian/ Native American	0.139	1.114	0.125	9.01E-01	-2.045	2.323	1.149	0.129	10.210	1.00E+00	-3.472	3.751	0.031	42.563
Multiracial	-0.843	1.036	-0.814	4.16E-01	-2.874	1.188	0.430	0.056	3.280	1.00E+00	-4.202	2.515	0.015	12.371
Gender: Male	0.096	0.254	0.379	7.05E-01	-0.402	0.594	1.101	0.669	1.812	1.00E+00	-0.728	0.920	0.483	2.510
BMI	0.073	0.020	3.635	2.78E-04	0.034	0.112	1.076	1.034	1.119	1.17E-02	0.008	0.138	1.008	1.148
Null Deviance (df)	654.25 (1509)													
Residual Deviance (df)	593.65 (1498)													
Observations deleted due to missingness	9													
AIC	617.650													

© 2019 Rosoff DB et al. *JAMA Network Open.*

Hoslem-Lemeshow Goodness of Fit: P-value	> 0.99													
										**Adjustment: Bonferroni method for 42 comparisons**				
**Outcome: Clinically High Total Cholesterol**	**Estimate**	**SE**	**Z**	**Pr(>|z|)**	**LCI**	**UCI**	**OR**	**OR LCI**	**OR UCI**	**P value**	**LCI**	**UCI**	**OR LCI**	**OR LCI**
(Intercept)	-5.085	0.596	-8.525	2.00E-16	-6.254	-3.916	0.006	0.002	0.020	8.40E-15	-7.018	-3.152	0.001	0.043
Count of days drinking at level I	0.019	0.005	3.806	1.41E-04	0.009	0.029	1.020	1.009	1.030	5.92E-03	0.003	0.036	1.003	1.036
Count of days drinking at level II	0.023	0.005	4.32	1.56E-05	0.013	0.034	1.024	1.013	1.035	6.55E-04	0.006	0.041	1.006	1.042
Count of days drinking at level III	0.031	0.008	3.934	8.35E-05	0.016	0.046	1.031	1.016	1.047	3.51E-03	0.005	0.056	1.005	1.058
Total Drinks	-0.001	0.000	-1.909	5.63E-02	-0.001	0.000	0.999	0.999	1.000	1.00E+00	-0.002	0.000	0.998	1.000
Age at admission	0.034	0.008	4.164	3.13E-05	0.018	0.051	1.035	1.018	1.052	1.31E-03	0.008	0.061	1.008	1.063
Race: Black/ African-American	-0.500	0.190	-2.633	8.47E-03	-0.873	-0.128	0.606	0.418	0.880	3.56E-01	-1.116	0.116	0.328	1.123
Race: Asian	-0.396	0.748	-0.529	5.97E-01	-1.861	1.069	0.673	0.156	2.914	1.00E+00	-2.819	2.027	0.060	7.592
Race: American Indian/ Native American	-0.446	1.115	-0.399	6.90E-01	-2.632	1.741	0.640	0.072	5.702	1.00E+00	-4.061	3.170	0.017	23.804
Multiracial	-0.840	0.752	-1.117	2.64E-01	-2.314	0.634	0.432	0.099	1.885	1.00E+00	-3.277	1.597	0.038	4.939
Gender: Male	-0.167	0.195	-0.858	3.91E-01	-0.549	0.215	0.846	0.577	1.239	1.00E+00	-0.799	0.464	0.450	1.591
BMI	0.034	0.017	2.042	4.11E-02	0.001	0.067	1.035	1.001	1.070	1.00E+00	-0.020	0.089	0.980	1.093
Null Deviance (df)	974.59 (1517)													
Residual Deviance (df)	879.8 (1506)													
Observations deleted due to missingness	1													
AIC	903.80													
Hoslem-Lemeshow Goodness of Fit: P-value	> 0.99													
										**Adjustment: Bonferroni method for 42 comparisons**				
**Outcome: Clinically High Triglycerides**	**Estimate**	**SE**	**Z**	**Pr(>|z|)**	**LCI**	**UCI**	**OR**	**OR LCI**	**OR UCI**	**P value**	**LCI**	**UCI**	**OR LCI**	**OR LCI**
(Intercept)	-6.256	0.632	-9.899	2.00E-16	-7.495	-5.017	0.002	0.001	0.007	8.40E-15	-8.305	-4.208	0.000	0.015
Count of days drinking at level I	0.018	0.005	3.383	7.16E-04	0.007	0.028	1.018	1.007	1.028	3.01E-02	0.001	0.035	1.001	1.035
Count of days drinking at level II	0.015	0.006	2.615	8.93E-03	0.004	0.026	1.015	1.004	1.026	3.75E-01	-0.003	0.033	0.997	1.033
Count of days drinking at level III	0.018	0.008	2.356	1.85E-02	0.003	0.034	1.019	1.003	1.034	7.77E-01	-0.007	0.044	0.993	1.045
Total Drinks	0.000	0.000	-0.858	3.91E-01	-0.001	0.000	1.000	0.999	1.000	1.00E+00	-0.001	0.001	0.999	1.001
Age at admission	0.005	0.008	0.574	5.66E-01	-0.012	0.021	1.005	0.988	1.022	1.00E+00	-0.023	0.032	0.978	1.033
Race: Black/ African-American	-0.850	0.209	-4.074	4.62E-05	-1.259	-0.441	0.427	0.284	0.643	1.94E-03	-1.527	-0.174	0.217	0.840
Race: Asian	0.125	0.561	0.222	8.24E-01	-0.974	1.223	1.133	0.377	3.399	1.00E+00	-1.692	1.942	0.184	6.970
Race: American Indian/ Native American	0.258	1.118	0.23	8.18E-01	-1.934	2.449	1.294	0.145	11.573	1.00E+00	-3.366	3.881	0.035	48.469
Multiracial	-0.861	0.750	-1.148	2.51E-01	-2.332	0.609	0.423	0.097	1.839	1.00E+00	-3.294	1.571	0.037	4.810
Gender: Male	0.687	0.229	3	2.70E-03	0.238	1.136	1.988	1.269	3.114	1.14E-01	-0.055	1.429	0.946	4.175
BMI	0.105	0.017	6.196	5.79E-10	0.072	0.139	1.111	1.075	1.149	2.43E-08	0.050	0.160	1.051	1.174
Null Deviance (df)	901.83 (1518)													
Residual Deviance (df)	815.27 (1507)													
Observations deleted due to missingness	0													
AIC	839.30													
Hoslem-Lemeshow Goodness of Fit: P-value	> 0.99													
										**Adjustment: Bonferroni method for 42 comparisons**				
**Outcome: Clinically High ALT**	**Estimate**	**SE**	**Z**	**Pr(>|z|)**	**LCI**	**UCI**	**OR**	**OR LCI**	**OR UCI**	**P value**	**LCI**	**UCI**	**OR LCI**	**OR LCI**

© 2019 Rosoff DB et al. *JAMA Network Open.*

(Intercept)	-2.209	0.361	-6.123	9.17E-10	-2.917	-1.502	0.110	0.054	0.223	3.85E-08	-3.379	-1.040	0.034	0.353
Count of days drinking at level I	0.019	0.003	5.701	1.19E-08	0.013	0.026	1.019	1.013	1.026	5.00E-07	0.008	0.030	1.008	1.031
Count of days drinking at level II	0.019	0.004	5.524	3.31E-08	0.013	0.026	1.020	1.013	1.027	1.39E-06	0.008	0.031	1.008	1.031
Count of days drinking at level III	0.028	0.005	5.389	7.08E-08	0.018	0.038	1.028	1.018	1.038	2.97E-06	0.011	0.044	1.011	1.045
Total Drinks	0.000	0.000	-0.657	5.11E-01	-0.001	0.000	1.000	0.999	1.000	1.00E+00	-0.001	0.001	0.999	1.001
Age at admission	0.011	0.005	2.143	3.21E-02	0.001	0.021	1.011	1.001	1.022	1.00E+00	-0.006	0.028	0.994	1.028
Race: Black/ African-American	-0.858	0.126	-6.831	8.41E-12	-1.104	-0.612	0.424	0.332	0.542	3.53E-10	-1.265	-0.451	0.282	0.637
Race: Asian	-0.103	0.343	-0.301	7.64E-01	-0.777	0.570	0.902	0.460	1.768	1.00E+00	-1.217	1.010	0.296	2.745
Race: American Indian/ Native American	-1.130	0.875	-1.291	1.97E-01	-2.846	0.586	0.323	0.058	1.796	1.00E+00	-3.967	1.707	0.019	5.513
Multiracial	-0.433	0.381	-1.138	2.55E-01	-1.180	0.313	0.648	0.307	1.368	1.00E+00	-1.668	0.801	0.189	2.228
Gender: Male	-0.393	0.128	-3.078	2.08E-03	-0.643	-0.143	0.675	0.526	0.867	8.74E-02	-0.806	0.021	0.447	1.021
BMI	0.032	0.012	2.738	6.18E-03	0.009	0.054	1.032	1.009	1.056	2.60E-01	-0.006	0.069	0.994	1.072
Null Deviance (df)	1983 (1518)													
Residual Deviance (df)	1732.3 (1507)													
Observations deleted due to missingness	0													
AIC	1756.30													
Hoslem-Lemeshow Goodness of Fit: P-value	> 0.99													
										**Adjustment: Bonferroni method for 42 comparisons**				
**Outcome: Clinically High AST**	**Estimate**	**SE**	**Z**	**Pr(>|z|)**	**LCI**	**UCI**	**OR**	**OR LCI**	**OR UCI**	**P value**	**LCI**	**UCI**	**OR LCI**	**OR LCI**
(Intercept)	-2.838	0.429	-6.617	3.67E-11	-3.678	-1.997	0.059	0.025	0.136	1.54E-09	-4.228	-1.448	0.015	0.235
Count of days drinking at level I	0.026	0.004	7.262	3.80E-13	0.019	0.033	1.027	1.019	1.034	1.60E-11	0.015	0.038	1.015	1.039
Count of days drinking at level II	0.027	0.004	7.365	1.77E-13	0.020	0.035	1.028	1.020	1.035	7.43E-12	0.015	0.039	1.015	1.040
Count of days drinking at level III	0.035	0.005	6.609	3.87E-11	0.024	0.045	1.035	1.025	1.046	1.63E-09	0.018	0.052	1.018	1.053
Total Drinks	0.000	0.000	-1.397	1.63E-01	-0.001	0.000	1.000	0.999	1.000	1.00E+00	-0.001	0.000	0.999	1.000
Age at admission	0.032	0.006	5.443	5.24E-08	0.020	0.043	1.032	1.020	1.044	2.20E-06	0.013	0.050	1.013	1.052
Race: Black/ African-American	-0.918	0.138	-6.651	2.92E-11	-1.188	-0.647	0.399	0.305	0.524	1.23E-09	-1.365	-0.470	0.255	0.625
Race: Asian	-0.454	0.467	-0.972	3.31E-01	-1.369	0.461	0.635	0.254	1.586	1.00E+00	-1.967	1.059	0.140	2.884
Race: American Indian/ Native American	-0.705	0.894	-0.788	4.31E-01	-2.458	1.048	0.494	0.086	2.853	1.00E+00	-3.604	2.194	0.027	8.973
Multiracial	-0.847	0.470	-1.803	7.13E-02	-1.767	0.074	0.429	0.171	1.076	1.00E+00	-2.369	0.675	0.094	1.965
Gender: Male	0.533	0.148	3.616	2.99E-04	0.244	0.823	1.705	1.277	2.276	1.26E-02	0.055	1.012	1.057	2.750
BMI	-0.023	0.013	-1.704	8.84E-02	-0.049	0.003	0.977	0.952	1.003	1.00E+00	-0.067	0.021	0.935	1.021
Null Deviance (df)	1825.3 (1516)													
Residual Deviance (df)	1462.9 (1505)													
Observations deleted due to missingness	2													
AIC	1486.90													
Hoslem-Lemeshow Goodness of Fit: P-value	> 0.99													
										**Adjustment: Bonferroni method for 42 comparisons**				
**Outcome: Clinically High GGT**	**Estimate**	**SE**	**Z**	**Pr(>|z|)**	**LCI**	**UCI**	**OR**	**OR LCI**	**OR UCI**	**P value**	**LCI**	**UCI**	**OR LCI**	**OR LCI**
(Intercept)	-4.207	0.404	-10.414	2.00E-16	-4.999	-3.415	0.015	0.007	0.033	8.40E-15	-5.516	-2.898	0.004	0.055
Count of days drinking at level I	0.022	0.004	5.661	1.50E-08	0.014	0.029	1.022	1.014	1.030	6.30E-07	0.009	0.034	1.009	1.035
Count of days drinking at level II	0.027	0.004	6.041	1.53E-09	0.018	0.036	1.028	1.019	1.037	6.43E-08	0.013	0.042	1.013	1.043
Count of days drinking at level III	0.032	0.007	4.657	3.20E-06	0.019	0.046	1.033	1.019	1.047	1.34E-04	0.010	0.054	1.010	1.056

© 2019 Rosoff DB et al. *JAMA Network Open.*

Total Drinks	0.000	0.000	-0.181	8.57E-01	-0.001	0.001	1.000	0.999	1.001	1.00E+00	-0.001	0.001	0.999	1.001
Age at admission	0.033	0.005	6.052	1.43E-09	0.022	0.043	1.033	1.022	1.044	6.01E-08	0.015	0.050	1.015	1.051
Race: Black/ African-American	0.188	0.131	1.443	1.49E-01	-0.067	0.444	1.207	0.935	1.559	1.00E+00	-0.235	0.611	0.791	1.843
Race: Asian	0.004	0.361	0.012	9.90E-01	-0.703	0.712	1.004	0.495	2.038	1.00E+00	-1.166	1.175	0.312	3.237
Race: American Indian/ Native American	-0.365	0.840	-0.434	6.64E-01	-2.010	1.281	0.695	0.134	3.600	1.00E+00	-3.085	2.356	0.046	10.553
Multiracial	-0.261	0.398	-0.657	5.11E-01	-1.041	0.518	0.770	0.353	1.679	1.00E+00	-1.551	1.028	0.212	2.796
Gender: Male	0.879	0.133	6.601	4.08E-11	0.618	1.140	2.408	1.855	3.127	1.71E-09	0.447	1.311	1.564	3.709
BMI	0.057	0.013	4.48	7.46E-06	0.032	0.081	1.058	1.032	1.085	3.13E-04	0.016	0.098	1.016	1.102
Null Deviance (df)	2080.7 (1518)													
Residual Deviance (df)	1577.5 (1507)													
Observations deleted due to missingness	0													
AIC	1601.50													
Hoslem-Lemeshow Goodness of Fit: P-value	0.10													
NOTES:														
**Outcomes:**	**Type**	**Sex-specific**	**Definition**											
Clinically High HDL cholesterol	Dummy	no	1 if HDL-C ≥ 100 mg/dL, 0 otherwise											
Clinically High LDL cholesterol	Dummy	no	1 if LDL-C ≥ 160 mg/dL, 0 otherwise											
Clinically High Total Cholesterol	Dummy	no	1 if TC ≥ 240 mg/dL, 0 otherwise											
Clinically High Triglycerides	Dummy	no	1 if Triglycerides ≥ 200 mg/dL, 0 otherwise											
Clinically High ALT	Dummy	yes	Females: 1 if ALT ≥ 25 IU/L, 0 otherwise; Males: 1 if ALT ≥ 40 IU/L, 0 otherwise											
Clinically High AST	Dummy	no	1 if LDL-C ≥ 160 mg/dL, 0 otherwise											
Clinically High GGT	Dummy	no	1 if TC ≥ 240 mg/dL, 0 otherwise											
**Predictor:**	**Type**	**Sex-specific**	**Definition**											
Reference level: Count of days drinking at non-binge level	Continuous	yes	Days drinking at non-binge: for Females: < 4 standard drinks on average over days drinking, 0 otherwise; Males: < 5 standard drinks on average over days drinking, 0 otherwise											
Count of days drinking at level I	Continuous	yes	Days drinking at level I: for Females: [4,8) standard drinks on average over days drinking, 0 otherwise; Males: [5,10) standard drinks on average over days drinking, 0 otherwise											
Count of days drinking at level II	Continuous	yes	Days drinking at level II: for Females: [8,12) standard drinks on average over days drinking, 0 otherwise; Males: [10,15) standard drinks on average over days drinking, 0 otherwise											
Count of days drinking at level III	Continuous	yes	Days drinking at level II: for Females: ≥12 standard drinks on average over days drinking, 0 otherwise; Males: ≥15 standard drinks on average over days drinking, 0 otherwise											
**Covariates:**	**Type**	**Sex-specific**	**Definition**											
Total Drinks	Continuous	no	Number of standard drinks consumed in 90 days prior to screening/ admission per Time Line FollowBack											
Age	Continuous	no	Age at protocol screening or admission as in-patient if admitted											
Gender	Dummy	na	1 if male, 0 if female											
BMI	Continuous	no	body mass index											
Race	Dummy	no	1 if specified race, 0 otherwise											

© 2019 Rosoff DB et al. *JAMA Network Open.*

**Abbreviations:**														
CI Adjustment:	Bonferroni method for 21 tests with qnorm(1-(.05/2*21))=3.038													
P value adjustment:	Bonferroni method for 21 tests													
SE	standard error of estimate													
OR	odds ratio (since estimate is ln(OR), OR=EXP(estimate))													
LCI	lower confidence interval bound													
UCI	upper confidence interval bound													

© 2019 Rosoff DB et al. *JAMA Network Open.*
